# Deciphering Cancer-Specific H3K27ac Landscapes Through Systematic Multi-Omics Integration and Development of Open-Access Genomic Annotation Platform

**DOI:** 10.3390/ijms27219606

**Published:** 2026-10-27

**Authors:** Yu-Shuo Zhuang, Yuan-Yuan Gao, Zhao-Yue Zhang, Zhe-Peng Wang, Jun-Jie Liu, Li-Rong Zhang, Siqin Bateer, Lu-Qiang Zhang

**Affiliations:** 1 Inner Mongolia Key Laboratory of Biophysics and Bioinformatics, School of Physical Science and Technology, Inner Mongolia University, Hohhot 010021, China; 13734783903@163.com (Y.-S.Z.); tr3135582094@163.com (Z.-P.W.); pyljj@imu.edu.cn (J.-J.L.); pyzlr@imu.edu.cn (L.-R.Z.); 2 Inner Mongolia Traditional Chinese & Mongolian Medical Research Institute, Hohhot 010020, China; 15849324447@163.com; 3 School of Medicine, University of Electronic Science and Technology of China, Chengdu 610054, China; zyzhang@uestc.edu.cn

**Keywords:** H3K27ac, cancer-specific patterns, multi-omics analyses, annotation resource

## Abstract

H3K27ac epigenetically modulates gene expression and serves as an indicator of cancer status. Although aberrant H3K27ac has been widely studied, a comprehensive landscape of cancer-specific H3K27ac patterns remains lacking. By integrating 209 H3K27ac ChIP-seq datasets across 48 tissues and cell types, we defined 3612 universally conserved regions and a vast repertoire of cancer-specific H3K27ac alterations. Strikingly, while cancer-specific H3K27ac gains were pervasive across leukemia, hepatocellular carcinoma, breast cancer, lung adenocarcinoma, and lymphoma, losses were remarkably rare. These cancer-specific H3K27ac gains were predominantly enriched in non-coding regions, where they were associated with increased chromatin accessibility and chromatin interactions, enabling the recruitment of oncogenic transcription factors to activate target genes. Conversely, cancer-specific H3K27ac losses preferentially occurred in promoters and correlated with chromatin compaction and transcriptional repression. Mechanistic multi-omics analyses in leukemia further demonstrated this epigenetic-structural coupling. Leukemia-specific H3K27ac gains established a permissive chromatin environment that facilitated oncogenic GATA1 binding and activation of its target gene TAL1. Within this context, TAL1 exhibited increased interaction with STIL, a well-known fusion partner implicated in leukemogenesis. To enable broader application of these findings, we developed an open-access web server for genomic annotation. Collectively, our study provides a comprehensive landscape of cancer-specific H3K27ac modifications, defining them as a functional epigenetic signature of oncogenic chromatin remodeling and offering an annotation resource for understanding the regulation of the cancer genome.

## 1. Background

According to the World Health Organization’s 2024 report, over 20 million new cancer cases and 9.7 million cancer-related deaths occurred worldwide in 2022. Approximately 53.5 million individuals had been diagnosed with cancer within the past five years and were still alive. In China, an estimated 4.82 million new cancer cases and 2.57 million cancer-related deaths occurred in 2022 [1]. This burden highlights the urgent need for a deeper understanding of the molecular drivers behind malignant transformation.

Cancer is a multifactorial disease arising from intricate interactions among genetic variation, epigenetic modifications, and environmental influences [1,2,3,4]. Among these, epigenetic dysregulation serves as a critical mechanism bridging genetic variation and environmental exposure [5,6,7]. Their perturbations can lead to inappropriate activation of oncogenes or silencing of tumor suppressors. Recent studies have demonstrated that distinct combinations of histone modifications (HMs) at promoters, enhancers, and gene bodies define disease-relevant chromatin states and contribute to cancer subtype heterogeneity [8,9,10,11,12,13].

Among various HMs, the acetylation of histone H3 at lysine 27 (H3K27ac) is a well-recognized indicator of active chromatin. By neutralizing histone tail charges, H3K27ac reduces nucleosome DNA affinity, increasing chromatin accessibility for transcription factor (TF) binding [14]. Numerous studies have linked aberrant H3K27ac patterns to oncogene activation in diverse cancers. For example, in breast cancer (BRCA), NBR2 dynamically regulates H3K27ac modifications at enhancer regions, activating TBX1 expression and promoting M2 macrophage polarization, which facilitates tumor metastasis [15]. In colorectal cancer, H3K27ac-mediated activation of STEAP3 leads to the formation of a complex with ATF3, which promotes tumor cell proliferation and migration [16]. Genome-wide H3K27ac analysis by Zhou et al. and Cai et al. identified super-enhancers and their related oncogenic targets, illuminating how H3K27ac shapes oncogenic transcriptional landscapes [17,18]. Gopi et al. revealed that among key HMs, H3K27ac exhibits markedly more enrichment peaks than H3K4me3, H3K9me3, and H4K20me3 across the NCI-60 panel of cancer cell lines [19]. However, these studies focused on differential H3K27ac regions during oncogenesis, paying limited attention to cancer-specific H3K27ac modification events that occur only in a particular cancer type and are absent in other cancers and normal samples. Moreover, how these modifications affect chromatin structure and transcription remains largely uncharacterized.

To address this gap, we curated 209 H3K27ac ChIP-seq datasets and systematically identified a core set of highly conserved H3K27ac modification regions shared across the majority of samples, as well as cancer-specific H3K27ac alteration regions for each studied cancer type. By integrating complementary multi-omics data including ChIP-seq data for histone modifications and transcription factors, RNA-seq, ATAC-seq, DNase-seq, and Hi-C data, we characterized genomic and epigenomic features of these regions and validated these findings in a leukemia model. To facilitate broad use, we developed a publicly accessible web server for rapid annotation of user-specified genomic regions. Collectively, our study suggests that cancer-specific H3K27ac modification is a permissive hallmark facilitating oncogenic transcription.

## 2. Results

### 2.1. Genome-Wide Identification and Characterization of Conserved and Cancer-Specific H3K27ac Patterns

#### 2.1.1. Global H3K27ac Profiling Defines a Core Set of Conserved H3K27ac-Modified Regions

**Figure 1:**
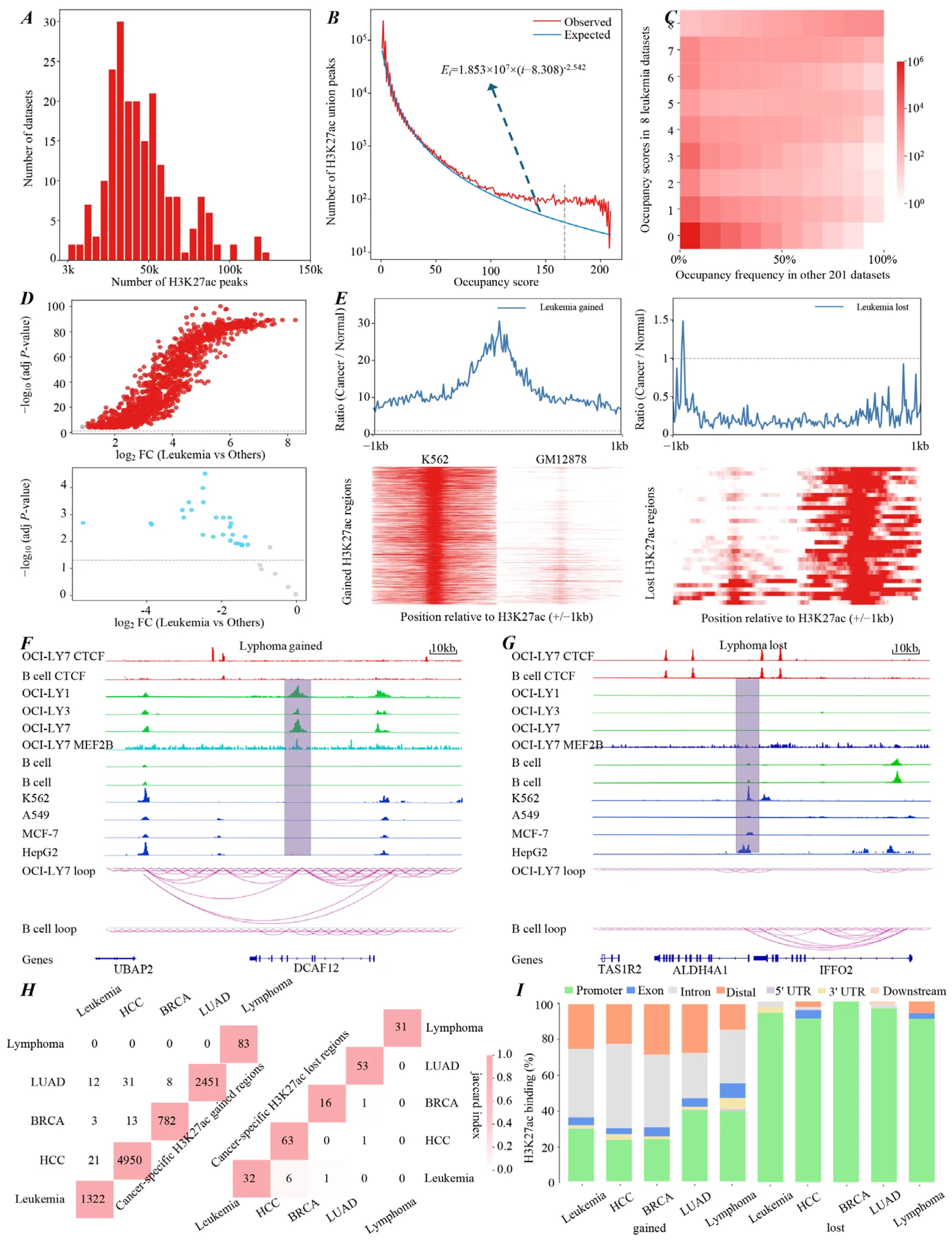
Global landscape and characterization of conserved and cancer-specific H3K27ac regions. (**A**) Global distribution of H3K27ac peaks across 209 ChIP-seq datasets. (**B**) Observed versus power-law model-predicted distributions of H3K27ac occupancy scores for 678,446 union peaks. (**C**) Occupancy score distribution of union peaks in eight leukemia datasets (y-axis) versus their occupancy frequencies across all other samples (x-axis). Color density reflects union peak number. (**D**) H3K27ac signal enrichment in leukemia samples for gained (**top**) and lost (**bottom**) regions relative to all other samples (dashed line *p*-value = 0.05). (**E**) H3K27ac ChIP-seq signal intensities (±1 kb from peak center) in leukemia K562 cells and normal GM12878 cells. The line plots (upper panels) show the average ratios of leukemia to normal signal intensities, with the dashed line representing a ratio of 1. (**F**,**G**) Genome browser tracks and chromatin loop diagrams of representative lymphoma-specific H3K27ac gained and lost regions. (**H**) Number and pairwise overlap of cancer-specific H3K27ac gained (**left**) and lost (**right**) regions across five cancer types. Color scale represents Jaccard similarity, with darker shades indicating greater overlap. (**I**) Genomic distribution of cancer-specific gained (**left**) and lost (**right**) H3K27ac regions across the five cancer types.

We analyzed 209 publicly available H3K27ac ChIP-seq datasets. Peak calling yielded a total of 9,619,866 H3K27ac peaks across all samples, with a median of 41,192 peaks per dataset (Figure 1A). Analysis of the summit distances between adjacent high-quality H3K27ac peaks showed an inflection point at ~150 bp, reflecting a boundary to distinguish peaks belonging to the same region from those in different regions (Supplementary Figure S1A) [20]. Using this data-driven threshold (*l* = 150 bp), we merged overlapping or adjacent peaks into a set of 678,446 non-overlapping union H3K27ac regions (Supplementary Figure S1B).

We next quantified the occupancy score for each H3K27ac union peak (Supplementary Figure S1C). These scores exhibited a highly skewed distribution, where most union peaks were detected in only a few datasets, with their abundance decreasing sharply as occupancy scores increased (Figure 1B). Interestingly, a subset of union peaks showed strong conservation, appearing in the majority of datasets (e.g., peaks with occupancy scores > 150). To establish a reliable baseline for highly conserved H3K27ac-modified regions, we performed threshold sensitivity and cross-intersection analyses using occupancy score cutoffs of 70%, 75%, 80%, 85%, and 90% of the total datasets. The evaluation showed that the predefined threshold of *i* = 168 (corresponding to an occupancy score in ≥80% of datasets) yielded a stable set of conserved regions that was highly consistent with those obtained using alternative thresholds (Supplementary Figure S1D). Accordingly, the 80% occupancy threshold was selected as the optimal baseline for identifying universally active regulatory elements across diverse samples (Supplementary Table S1).

To further demonstrate that this conservation was not a stochastic artifact, we modeled the expected distribution of union peaks (*E_i_*) for a given occupancy score (*i*) using a power-law function and described as $E_{i}=1.853\times 10^{7}{\left(i-8.308\right)}^{-2.542}$ (Figure 1B). We then calculated the ratio of ${\sum_{i}^{209}O}_{i}$ to ${\sum_{i}^{209}E}_{i}$, where *O_i_* describes the number of observed H3K27ac union peaks with an occupancy score equal to *i*. Strikingly, at the predefined threshold (*i* = 168), the observed-to-expected ratio reached 3.26, indicating a robust enrichment of conserved H3K27ac regions beyond random chance.

#### 2.1.2. Widespread H3K27ac Profiling Reveals Cancer-Specific H3K27ac Landscapes

In contrast to the highly conserved regions described above, we next investigated whether cancer-specific H3K27ac alterations exist. To this end, we systematically compared H3K27ac occupancy scores and frequencies in each cancer type, including leukemia, hepatocellular carcinoma (HCC), BRCA, lung adenocarcinoma (LUAD), and lymphoma, with those in other samples and matched normal controls (Supplementary Table S2). We observed that some H3K27ac regions exhibited distinctive occupancy shifts. These regions showed significant enrichment or depletion in specific cancer types but opposite occupancy frequencies in all other samples (Figure 1C and Supplementary Figure S2A–D). Based on this observation, we systematically identified cancer-specific H3K27ac gained and lost regions using predefined occupancy and signal intensity thresholds (Supplementary Table S1; see Methods for details). The reliability of the predefined thresholds was evaluated by sensitivity and cross-intersection analyses following the same strategy used to identify conserved regions. The results showed that the cancer-specific H3K27ac gained and lost regions identified using the predefined thresholds were highly robust and consistent (Supplementary Figure S2E–G).

To further characterize H3K27ac patterns in these regions, we evaluated both the direction and magnitude of H3K27ac signal changes between cancer samples and all other sample types by comparing signal distribution and quantifying effect sizes. Signal distribution analyses showed that the gained regions exhibited significant H3K27ac deposition, whereas the lost regions exhibited marked H3K27ac depletion in specified cancer types relative to both matched normal controls and all other sample types (Figure 1D,E and Supplementary Figures S3 and S4A,B). For example, we identified 1322 gained and 32 lost regions in leukemia. The gained regions showed 5.55- to 30.64-fold increases in signal intensity relative to normal samples. In contrast, the lost regions showed a marked depletion, averaging only 0.24-fold of the signal intensities observed in matched normal samples (Figure 1E and Supplementary Figure S3D). Representative examples also included a lymphoma-specific gain at the DCAF12 promoter and a loss near the ALDH4A1 promoter (Figure 1F,G). The magnitude of these alterations was quantified through Cohen’s d effect sizes, where values of 0.2, 0.5, and ≥0.8 indicate small, medium, and large effects, respectively [21]. Notably, the absolute effect sizes for lost regions ranged from 0.59 to 0.95 (mean = 0.80), whereas gained regions exhibited markedly larger effect sizes of 2.05–3.88 (mean = 3.26; Supplementary Figure S3C). These results provide quantitative evidence that H3K27ac signal intensities in cancer-specific regions differ markedly in cancer samples compared with both matched normal controls and all other sample types.

#### 2.1.3. Cancer-Specific H3K27ac Regions Exhibit High Specificity and Spatial Preferences

To assess the extent of cancer-type specificity, we performed cross-cancer comparisons of the identified cancer-specific H3K27ac regions. Despite applying unified analytical frameworks, we observed minimal overlap of these regions among different cancer types (Figure 1H). This observation was further supported by Jaccard similarity index analysis, which quantitatively measures the degree of similarity between two sets [22]. Across the five cancer types, the average Jaccard indices were 0.002 for gained regions and 0.01 for lost regions (Figure 1H and Supplementary Table S3). In contrast, Jaccard indices for all H3K27ac regions across the same five cancer types ranged from 0.12 to 0.24, substantially higher than those observed for cancer-specific regions (Supplementary Figure S4C). These results collectively demonstrate the strong cancer-type specificity of the identified regions. Subsequently, we examined the genomic distribution of the identified cancer-specific H3K27ac regions. The analysis revealed distinct spatial preferences (Figure 1I and Supplementary Table S1). Specifically, gained regions were predominantly enriched in distal and non-coding genomic regions, particularly intronic, consistent with enhancer-associated features, suggesting a role in transcriptional reprogramming through distal regulatory elements. In contrast, lost regions were largely localized to promoter regions (>90% in all cancer types), indicating that lost H3K27ac regions may influence gene expression by altering promoter-associated chromatin states; the corresponding regions and annotated genes for each cancer type are listed in Supplementary Table S4. Collectively, these findings delineate a comprehensive landscape of H3K27ac modifications, encompassing both highly conserved and cancer-specific regions.

### 2.2. Cancer-Specific H3K27ac Modification Events Are Associated with Dynamic Changes in Chromatin Structure During Oncogenesis

#### 2.2.1. Cancer-Specific H3K27ac Modifications Accompany Local Chromatin State Alterations

**Figure 2:**
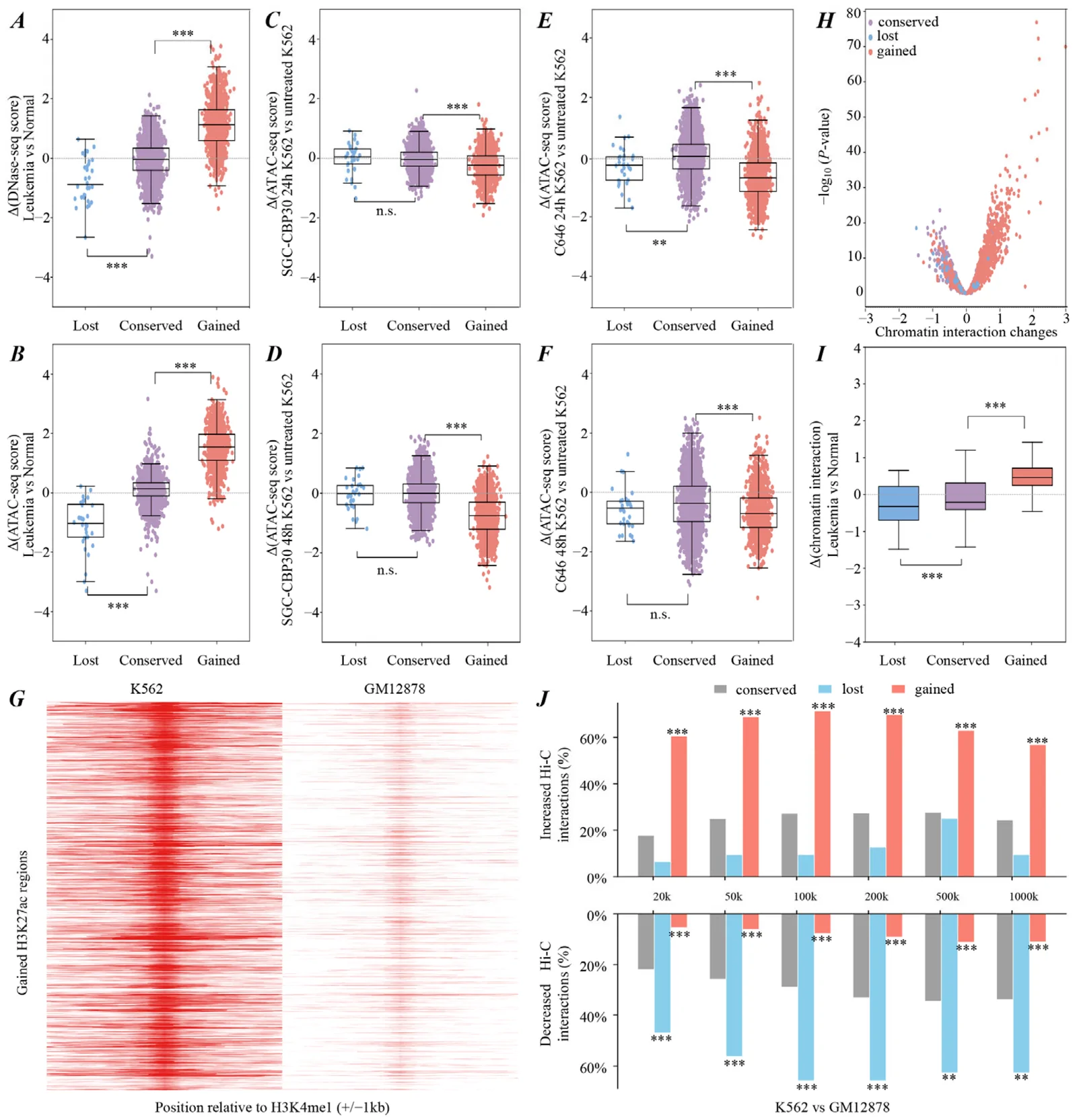
Cancer-specific H3K27ac alterations coordinate with chromatin accessibility and three-dimensional structure rewiring. (**A**,**B**) DNase-seq and ATAC-seq signal intensities at conserved and leukemia-specific gained and lost H3K27ac regions during leukemogenesis. (**C**–**F**) ATAC-seq signal intensity changes at the three types of H3K27ac regions within leukemia K562 cells following treatment with the CBP/p300 bromodomain inhibitor SGC-CBP30 (**C**,**D**) and the p300/CBP acetyltransferase inhibitor C646 (**E**,**F**). (**G**) H3K4me1 enrichment profiles centered at leukemia-specific gained H3K27ac regions in K562 and GM12878 cells. (**H**,**I**) Hi-C interaction profiles showing differential interaction frequencies at leukemia-specific H3K27ac regions compared with normal controls. (**J**) Proportions of H3K27ac regions exhibiting significant shifts in local chromatin interactions across multiple genomic distance scales. Per-region paired *t*-test *p*-values were adjusted using the Benjamini–Hochberg false discovery rate (FDR) method. Statistical significance was assessed using FDR-adjusted *p*-values (** *p*-value < 0.01, *** *p*-value < 0.001) n.s., not significant.

To explore whether cancer-specific H3K27ac alterations are associated with local chromatin state changes, we first quantified chromatin accessibility across 678,446 union H3K27ac regions using DNase-seq by Equation (1) (see Section 4.4) in leukemia, HCC, BRCA, LUAD, and lymphoma. Comparative analyses revealed that relative to normal samples, cancer-specific gained H3K27ac regions exhibited significantly elevated DNase-seq signals during oncogenesis. In contrast, lost regions showed markedly reduced accessibility, while conserved regions remained stable (Figure 2A and Supplementary Figure S5A–D). These findings were robustly validated using independent ATAC-seq datasets across leukemia, HCC, BRCA, and LUAD (Figure 2B and Supplementary Figure S5E–G). To functionally validate this epigenetic coupling, we analyzed publicly available ATAC-seq data from K562 cells treated with CBP/p300 bromodomain inhibitor SGC-CBP30 and acetyltransferase inhibitor C646, where CBP and p300 are the principal acetyltransferases responsible for acetylating histone H3 at lysine 27 [23,24]. In leukemia K562 cells, treatment with SGC-CBP30 and C646 led to a significant reduction in chromatin accessibility specifically at cancer-specific gained H3K27ac regions (Figure 2C–F). However, chromatin accessibility at conserved and cancer-specific lost regions remained relatively stable (Figure 2C,D), with reductions appearing only after 48 h of C646 treatment (Figure 2F).

#### 2.2.2. Cancer-Specific H3K27ac Gains and Losses Parallel Higher-Order Chromatin Remodeling

Beyond chromatin accessibility, we next investigated whether cancer-specific H3K27ac alterations are associated with higher-order chromatin remodeling. We first profiled the landscapes of CTCF and H3K4me1 at the identified cancer-specific H3K27ac alteration regions. Notably, CTCF binding was markedly elevated in the vicinity of cancer-specific gained H3K27ac regions (Figure 1F). H3K4me1 signal intensities also showed concordant changes with H3K27ac in both gained and lost regions across most cancers (Figure 2G and Supplementary Figure S5H–P). These observations suggest that cancer-specific H3K27ac gains or losses are coupled with the recruitment of architectural proteins and active enhancer markers.

To directly measure changes in higher-order chromatin structure, we analyzed Hi-C data across multiple cancers. Differential Hi-C contact maps across leukemia, BRCA, LUAD, and lymphoma revealed that H3K27ac gained or lost regions exhibited significantly increased or decreased interaction frequencies within their flanking regions relative to normal samples, respectively (Figure 2H,I and Supplementary Figure S6A,B). Representative examples included the lymphoma-specific H3K27ac gained region at the DCAF12 promoter and lost region near the ALDH4A1 promoter, where chromatin loop strengths were markedly increased and decreased, respectively, relative to normal control cells (Figure 1F,G). Even the effects of these H3K27ac alterations remained evident at extended genomic distances (Figure 2J and Supplementary Figure S6C). For example, in leukemia, 57% of gained regions maintained elevated interaction frequencies up to 1000 kb, while 62.5% of lost regions continued to show reduced chromatin contacts at this scale. Collectively, these findings indicate that cancer-specific H3K27ac gain and loss are accompanied by concordant remodeling of three-dimensional chromatin architecture.

### 2.3. Cancer-Specific H3K27ac Alterations Are Associated with Transcriptional Reprogramming, with Gained Regions Exhibiting Heightened Sensitivity to p300 Depletion

**Figure 3:**
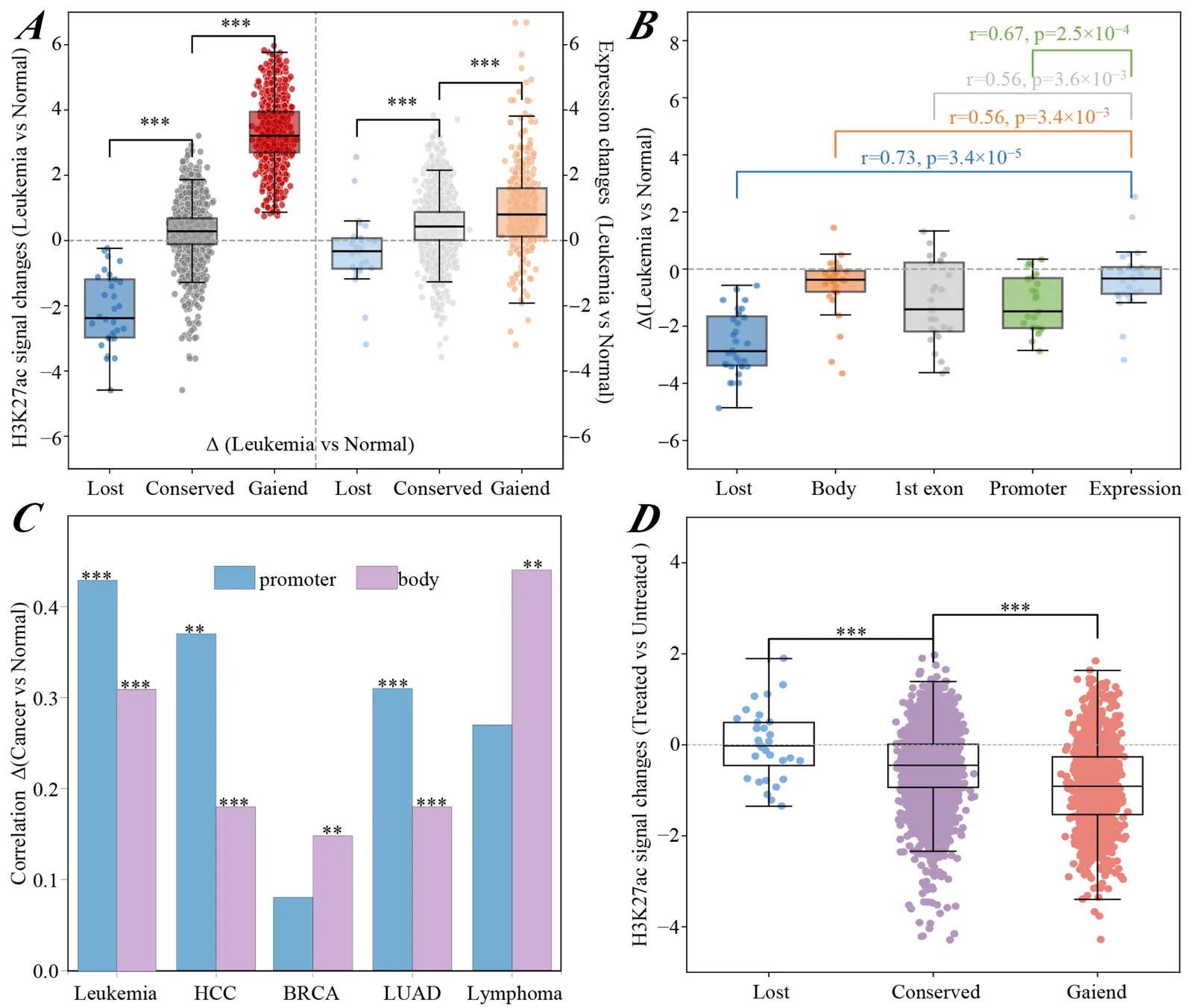
Cancer-specific H3K27ac modification events are associated with oncogenic transcriptional reprogramming. (**A**) Concordant shifts in H3K27ac signal intensities (**left**) and corresponding gene expression levels (**right**) across highly conserved and leukemia-specific gained and lost regions. (**B**) Correlation between H3K27ac signal intensity changes at different functional regions and corresponding gene expression changes during leukemogenesis. (**C**) Expression coupling between cancer-specific H3K27ac gained regions and their putative target genes. (**D**) H3K27ac signal intensity changes in distinct genomic regions within K562 cells after CRISPR-mediated p300 depletion relative to untreated K562 cells. Statistical significance was assessed by Wilcoxon rank-sum test (** *p*-value < 0.01, *** *p*-value < 0.001).

Recent studies revealed that chromatin structure changes functionally link to oncogenic reprogramming of transcriptional networks [25,26]. To explore the functional impact of H3K27ac alterations, we analyzed the correlation between H3K27ac signal changes and target gene expression changes. The results revealed that cancer-specific H3K27ac gains were linked to gene upregulation, while losses correlated with gene downregulation. In contrast, conserved regions displayed few changes in both acetylation and expression levels (Figure 3A and Supplementary Figure S7A–D).

We further benchmarked the regulatory potency of cancer-specific H3K27ac regions against canonical cis-regulatory elements, including promoters, gene bodies, and the first exons. Using Equation (1), we quantified H3K27ac signal intensities within these regions and calculated the Pearson correlation coefficients between acetylation changes and gene expression changes during oncogenesis. Remarkably, cancer-specific regions, particularly the lost regions, exhibited the highest correlations with gene expression changes (Figure 3B and Supplementary Figure S7E–H). These correlations significantly surpassed those of traditional regulatory elements. Gained regions showed stronger expression coupling with their putative promoter targets across multiple cancer types, including leukemia, HCC, and LUAD (Figure 3C).

We next explored the functional relevance of these H3K27ac modification regions by collecting and analyzing CRISPR-Cas9-mediated p300 depletion data. Notably, cancer-specific gained regions were more sensitive to p300 loss than conserved regions. These gained regions exhibited a marked depletion of H3K27ac signals following p300 depletion. However, cancer-specific lost regions remained largely unaffected (Figure 3D). These findings suggest that cancer-specific H3K27ac modifications may be closely associated with transcriptional reprogramming, exhibiting marked sensitivity to p300 depletion and pharmacological inhibition of histone acetyltransferase activity.

### 2.4. Cancer-Specific H3K27ac Gains May Facilitate Oncogenic Transcription Factor Recruitment and Cancer-Associated Transcriptional Reprogramming

#### 2.4.1. Cancer-Specific H3K27ac Gained Regions May Facilitate Oncogenic TF Recruitment

**Figure 4:**
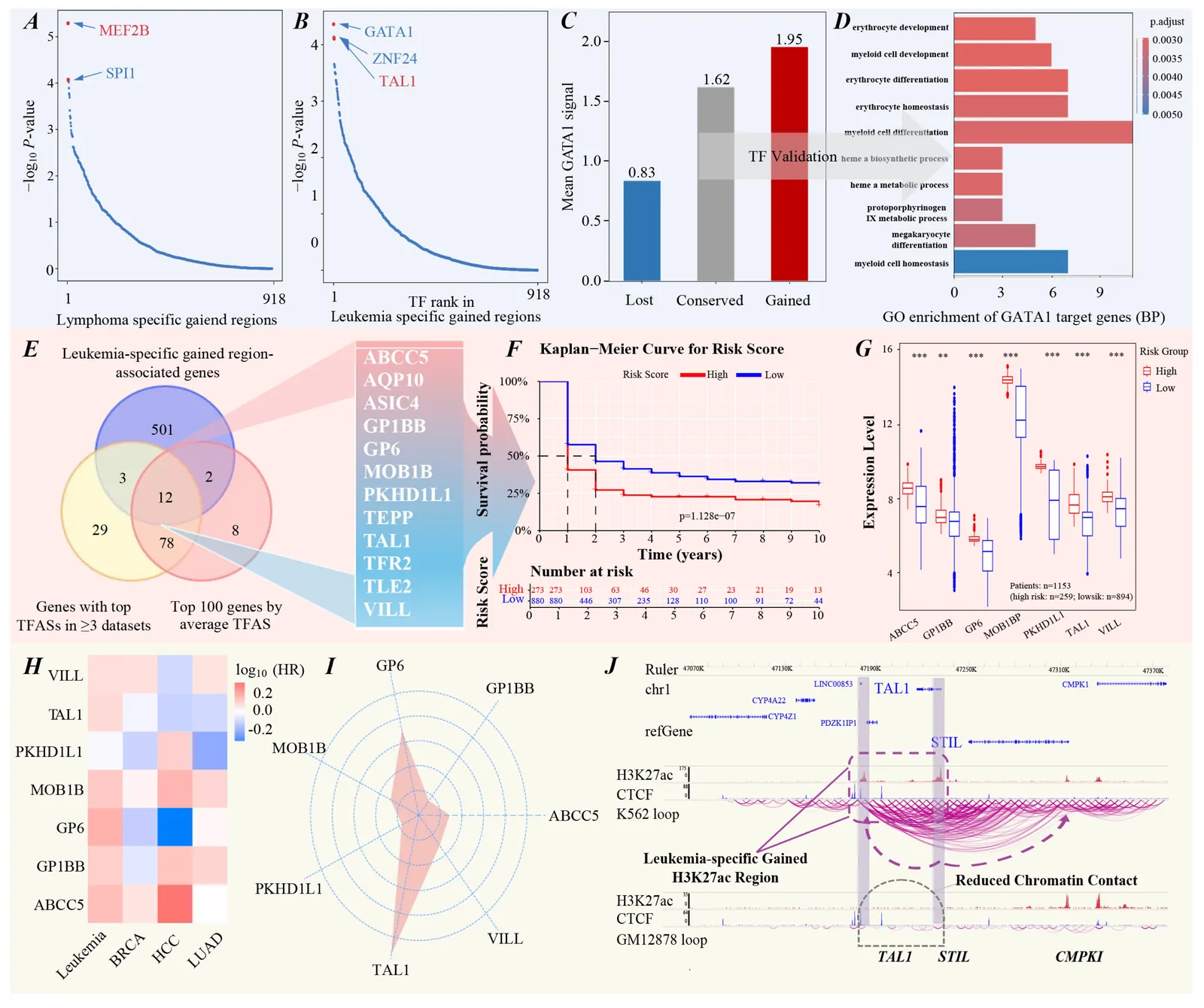
Cancer-specific H3K27ac gains are associated with oncogenic transcription factor recruitment and transcriptional dysregulation. (**A**,**B**) TFs predicted by BART to be enriched in lymphoma-specific and leukemia-specific gained H3K27ac regions. (**C**) Average GATA1 signal intensities at conserved regions and leukemia-specific gained and lost H3K27ac regions in leukemia K562 cells. (**D**) GO biological process enrichment analysis of GATA1 target genes. (**E**) Analytical framework for identifying genes that are both located in leukemia-specific gained H3K27ac regions and regulated by GATA1. (**F**) Kaplan–Meier survival curves stratifying leukemia patients into high- and low-risk groups. (**G**) Expression level distributions of the seven identified genes across different leukemia risk-stratified samples (** *p*-value < 0.01, *** *p*-value < 0.001). (**H**) Pan-cancer HR analysis of the 7 identified genes across leukemia, BRCA, HCC, and LUAD. (**I**) Regulatory weights of GATA1 on the seven identified genes calculated by GENIE3 algorithm. (**J**) Genome browser snapshots of the TAL1 locus in leukemia K562 cells and normal GM12878 cells. Purple bars denote leukemia-specific gained H3K27ac regions and purple lines indicate chromatin interaction loops identified by Hi-C analysis.

H3K27ac is widely recognized as a hallmark of active chromatin, mainly enriched at promoters and enhancers [25]. These regions usually recruit oncogenic TFs to drive tumorigenesis [27,28]. Our analyses showed that cancer-specific H3K27ac modification regions were associated with changes in chromatin structure and gene expression. We therefore hypothesized that these alterations may contribute to a permissive chromatin environment associated with selective oncogenic TF recruitment and transcriptional network reorganization during malignant transformation. Using the BART algorithm [29], we inferred TF enrichment in these cancer-specific modification regions across leukemia, HCC, BRCA, LUAD, and lymphoma. The top two enriched TFs in these regions and their enrichment scores are depicted in Figure 4A,B, Supplementary Figure S8A–H, and Table S5. The identified TFs exhibited high functional relevance to their respective cancers. For example, MEF2B was the most significantly enriched TF in lymphoma-specific gained regions (Figure 4A), and these regions exhibited increased MEF2B binding signal intensities (Figure 1F). This finding aligns with the well-established roles of MEF2B in multiple lymphoma subtypes [30]. Conversely, KDM5C emerged as the most enriched factor within lymphoma-specific lost H3K27ac regions (Supplementary Figure S8H), which can downregulate p53 expression and promote diffuse large B-cell lymphoma progression [31]. We also identified TFs with cross-cancer enrichment patterns, with BRD4 as a notable case. BRD4 ranked prominently in the lost H3K27ac regions of leukemia, HCC, and LUAD (Supplementary Figure S8D,E,G), indicating an association between BRD4 occupancy and regions with reduced H3K27ac in these cancers. The functional and therapeutic significance of this association remains to be determined.

#### 2.4.2. The GATA1-TAL1 Axis May Drive Leukemogenesis and Predict Patient Survival

To validate the functional impact of these TF recruitments, we focused on GATA1, a zinc finger TF that showed the greatest enrichment in leukemia-specific gained H3K27ac regions (Figure 4B). ChIP-seq analysis revealed that GATA1 binding signal intensity was 1.20-fold and 2.35-fold higher in leukemia-specific gained regions than in conserved and lost regions (Figure 4C), consistent with its enrichment in leukemia-specific gained H3K27ac regions. GO analysis revealed that GATA1 target genes were primarily involved in erythropoiesis and myeloid lineage development and regulation (Figure 4D), which are central to leukemia pathophysiology.

By intersecting GATA1 targets with genes linked to leukemia-specific gained H3K27ac regions, we identified 12 genes that are both located in leukemia-specific gained H3K27ac regions and regulated by GATA1 (Figure 4E and Supplementary Table S6). Based on our previously developed clinical risk stratification framework [32], we performed 1000 iterations of LASSO-Cox regression with 10-fold cross-validation and retained 7 of the 12 genes that had nonzero coefficients in at least 95% of iterations. These genes significantly classified 1153 leukemia patients into high- and low-risk groups based on the optimal risk score cutoff (Figure 4F), with higher expression observed in the high-risk group (n = 259) than in the low-risk group (n = 894) (Figure 4G). Similar results were observed in an independent TARGET cohort (Supplementary Figure S9A). Pan-cancer survival analysis [33] revealed that these genes exhibited markedly higher hazard ratios (HRs) in leukemia compared to other cancer types (Figure 4H). Among the seven genes, TAL1 was identified as the top-ranked GATA1-regulated gene based on GENIE3-derived regulatory weight [34] (Figure 4I). In leukemia cells, we identified two leukemia-specific gained H3K27ac regions near the TAL1 locus. Hi-C analysis revealed that these regions were associated with a more open chromatin environment and markedly enhanced spatial interactions between TAL1 and neighboring genes, including STIL (Figure 4J), which is involved in a recurrent rearrangement implicated in leukemogenesis [35]. This spatial restructuring may increase the physical proximity of the TAL1 and STIL loci and could potentially contribute to a genomic environment permissive for rearrangement; however, a direct causal relationship with STIL-TAL1 fusion formation remains to be experimentally established. The results were validated in an independent GSE152136 cohort (Supplementary Figure S9B). Together, these analyses suggest that cancer-specific H3K27ac gains are linked to a permissive chromatin environment that may facilitate oncogenic TF recruitment. Within this locally accessible and spatially restructured chromatin architecture, TF target loci may exhibit enhanced interactions with neighboring genomic regions, potentially contributing to oncogenic transcriptional reprogramming.

### 2.5. CCHMR: An Interactive Platform for Functional H3K27ac Annotation

**Figure 5:**
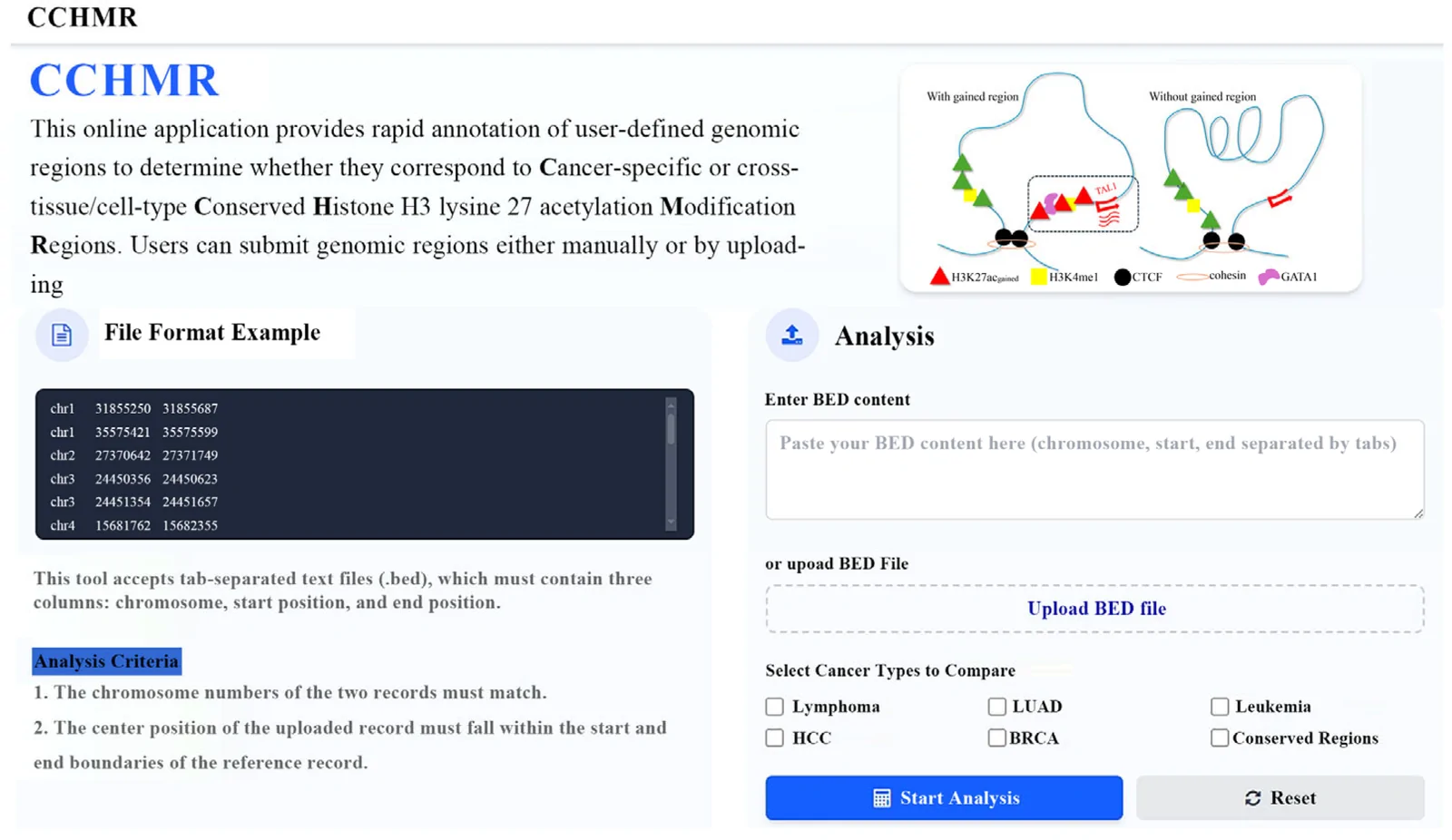
Interface and functional modules of the H3K27ac annotation web server.

To maximize the utility of our pan-cancer atlas for the research community, we developed CCHMR (Cancer-specific or cross-tissue/cell-type Conserved Histone H3 lysine 27 acetylation Modification Regions), a streamlined web server available at https://www.imu-zgroup.com/CCHMR/ (accessed on 20 October 2026). CCHMR offers several key features to facilitate rapid biological discovery (Figure 5 and Supplementary Figure S9C).

(1) Rapid functional annotation: Users can instantly determine whether their genomic regions of interest (e.g., potential enhancers or GWAS risk loci) overlap with the 3612 highly conserved H3K27ac regions or cancer-specific H3K27ac regions identified in this study.
(2) Intuitive interface: The server supports both manual entry and batch uploads of BED-formatted files, making it suitable for both single-locus queries and genome-wide screens.

## 3. Discussion

As a key component of epigenetic regulation, H3K27ac-mediated gene regulation contributes to pathogenesis not only in cancers but also in a wide range of other diseases. In Alzheimer’s disease, aberrant H3K27ac deposition has been linked to gene regulation implicated in neuroinflammation and synaptic dysfunction [36]. In myocardial infarction, H3K27ac promotes the expression of genes involved in cell proliferation, thereby facilitating cardiac repair [37]. These findings collectively highlight H3K27ac as a pivotal regulator of transcriptional programs across pathological states. However, these studies have largely focused on regions with differential H3K27ac signals during oncogenesis, regardless of disease-specific H3K27ac modification patterns and their underlying regulatory effects.

In this work, we addressed this gap by constructing a comprehensive, multi-cancer H3K27ac atlas, identifying cancer-specific H3K27ac modification regions in five distinct cancer types, as well as conserved regions across 48 cell and tissue types. By integrating multi-omics data including HM and TF ChIP-seq, RNA-seq, ATAC-seq, DNase-seq, and in situ Hi-C, we uncovered that cancer-specific H3K27ac modification events were associated with chromatin remodeling, altered chromatin accessibility and interactions, and oncogenic transcriptional programs. Specifically, cancer-specific H3K27ac gains are preferentially associated with increased chromatin accessibility and the formation of long-range chromatin interactions, often anchored by oncogenic TFs. These regions serve as transcriptionally permissive hubs, coordinating the activation of oncogenic programs. For example, in leukemia, we identified a GATA1-centered regulatory circuit wherein the oncogenic TF GATA1 is enriched at leukemia-specific gained H3K27ac modification regions (Figure 4B,C). Its target gene TAL1, a well-characterized driver in hematologic malignancies, is significantly upregulated in high-risk cases (Figure 4G). Notably, these regions exhibit increased chromatin loop formation, facilitating spatial proximity between TAL1 and nearby genes such as STIL (Figure 4J), a common partner in gene fusion events associated with leukemogenesis [35]. A schematic model illustrating the relationship between dynamic GATA1 binding, H3K27ac gain, and activation of the GATA1 target gene TAL1 in leukemia is shown in Figure 5 (top-right corner).

In contrast, cancer-specific lost H3K27ac events are primarily localized at promoters and associated with chromatin compaction and gene repression. Recent studies have hypothesized that H3K27ac enrichment upstream of promoters can serve as docking hubs for multiple distal enhancers, facilitating their coordinated looping to activate a single gene promoter [25,26]. In this regard, loss of such an H3K27ac docking region may impair multi-enhancer engagement, resulting in attenuated transcription. While our analysis suggests a consistent repressive outcome of H3K27ac loss, further mechanistic studies are warranted to establish causality between cancer-specific deacetylation and transcriptional silencing in cancer.

Nonetheless, our study has several limitations. First, this study is based primarily on the reanalysis of multi-source publicly available datasets, with perturbation-based validation mainly performed in leukemia. Therefore, differences in data quality, sequencing depth, sample size, and experimental design across the original studies may affect the consistency of the results. This issue may partly contribute to the substantial variation in the numbers of cancer-specific H3K27ac regions identified across the five cancer types. Although matched normal samples were incorporated to minimize tissue-of-origin effects, residual confounding arising from differences in tissue lineage, cell composition, experimental platform, and sample size across public datasets cannot be completely excluded. Second, our analyses focused on highly cancer-specific H3K27ac alterations, whereas broadly shared or less dynamic H3K27ac regions across cancers were not systematically investigated and may also have important regulatory functions. Third, although multiple omics layers were integrated to characterize chromatin accessibility, transcription, TF binding, and three-dimensional chromatin interactions, most analyses were performed at the bulk population level and may therefore obscure cell-type-specific regulatory heterogeneity. Finally, the associations identified between H3K27ac alterations, chromatin remodeling, and transcriptional regulation do not necessarily establish direct causality. Future studies integrating single-cell multi-omics, targeted functional perturbation, and in vivo validation will be required to further define the causal and cell-type-specific roles of cancer-associated H3K27ac remodeling.

## 4. Methods

### 4.1. Public Data Collection

**Figure 6:**
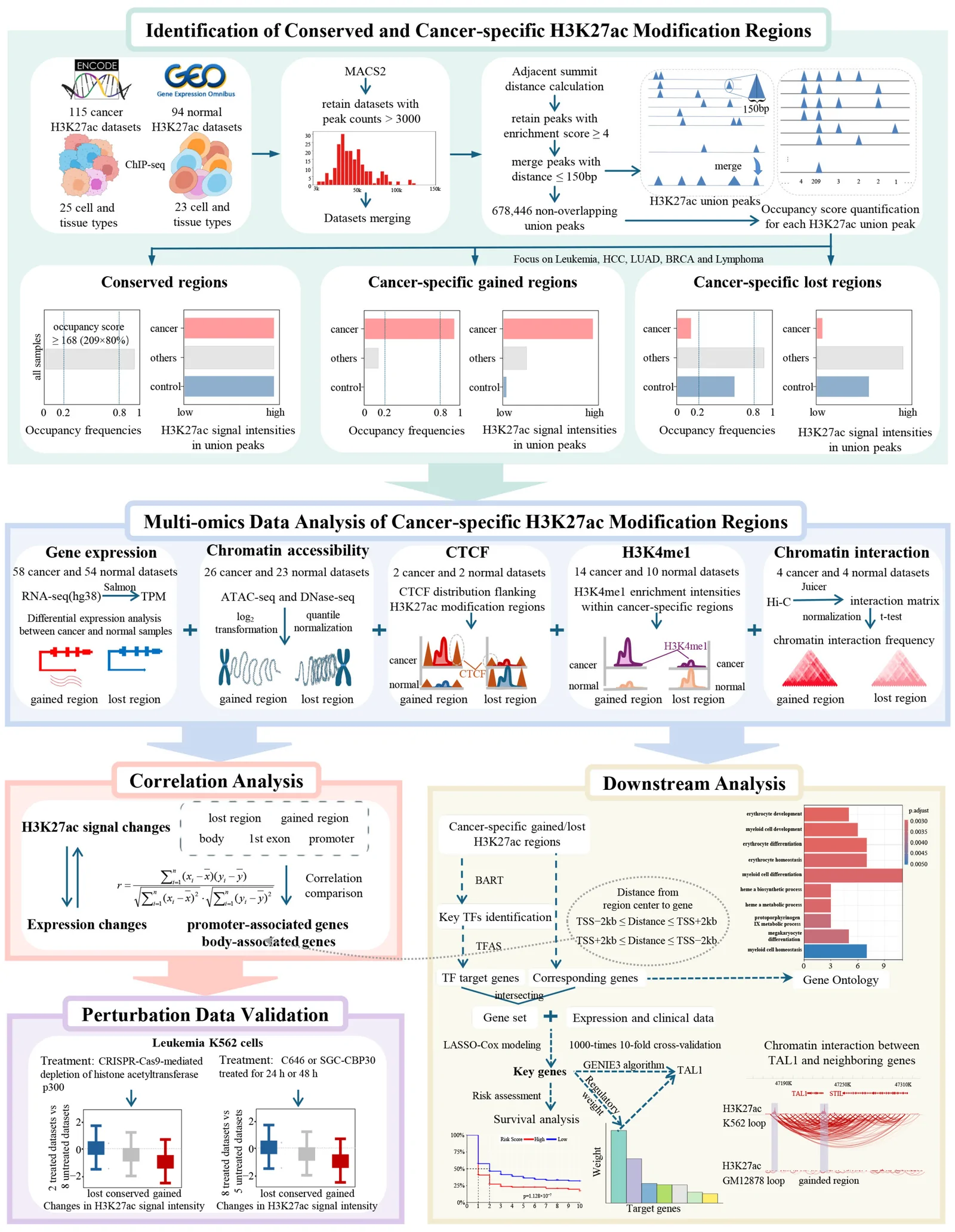
Schematic of the analytical pipeline for deciphering oncogenic chromatin remodeling and downstream regulatory circuitries.

An overview of the systematic framework and multi-omics integration strategies employed in this study is illustrated in Figure 6. In total, 209 H3K27ac and 24 H3K4me1 ChIP-seq datasets were obtained from the ENCODE and GEO databases, covering 48 and 10 distinct cell or tissue types across both healthy and malignant samples. To enable cross-referencing, we integrated these with matched multi-omics datasets, including 112 RNA-seq and 22 DNase-seq datasets for leukemia, HCC, LUAD, BRCA, and lymphoma cells and 10 RNA-seq datasets for leukemia patients; 27 ATAC-seq datasets for leukemia, HCC, BRCA, and LUAD; 8 Hi-C datasets for leukemia, BRCA, LUAD, and lymphoma cells; and 10 Hi-C datasets for leukemia patients. We additionally retrieved variable numbers of TF ChIP-seq datasets for GATA1, MEF2B, and CTCF to delineate the regulatory circuitries in leukemia and lymphoma.

To investigate the functional consequences of H3K27ac dysregulation, we collected perturbation-based leukemia datasets from public sources. This analysis included two H3K27ac ChIP-seq datasets generated following CRISPR-mediated p300 depletion, as well as eight ATAC-seq datasets from K562 cells treated with either the CBP/p300 bromodomain inhibitor SGC-CBP30 or the CBP/p300 acetyltransferase inhibitor C646 [23,24]. For clinical relevance and survival analysis, 9 gene expression cohorts with corresponding clinical information were collected from the GEO database and TARGET database. Normalized expression matrices were obtained for GSE71014 and TARGET data, while raw CEL files were downloaded and processed for the remaining 7 datasets. Detailed information for each dataset is comprehensively documented in Supplementary Table S7.

### 4.2. Data Preprocessing

#### 4.2.1. ChIP-Seq Data Processing and Standardized Pipeline

H3K27ac ChIP-seq datasets were available in BAM and FASTQ formats, while the H3K4me1 ChIP-seq datasets were provided only in BAM format. FASTQ-formatted ChIP-seq datasets were processed using the ENCODE4 ChIP-seq pipeline (v1.8.0; GRCh38) to ensure consistency [38]. Briefly, raw reads underwent quality control via FastQC [39]. High-quality reads were aligned to the human reference genome (hg38) using Bowtie2 [40]. The resulting SAM files were converted to BAM format, sorted and indexed using SAMtools [41]. PCR duplicates were removed using Picard [42]. For the generated H3K27ac BAM files, as well as the H3K27ac and GATA1 datasets obtained from the ENCODE database, peak calling was performed using MACS2 under a false discovery rate threshold of 0.01 [43]. Only H3K27ac samples with more than 3000 peaks were retained. For MEF2B, CTCF, and H3K27ac, BAM files were converted to BedGraph format using BEDTools and subsequently converted to BigWig format using bedGraphToBigWig [44] to facilitate visualization. Drawing on the RPKM formula, we calculated H3K27ac, H3K4me1, and GATA1 signal intensities using Equation (1) [45]. To systematically reduce technical batch effects and data heterogeneity across multi-source datasets, the log_2_-transformed signal values were subsequently subjected to quantile normalization, a rigorous, non-linear normalization approach [46]:

(1) $$S_{m}^{k}=\frac{N_{m}^{k}\times 10^{9}}{N_{m}\times L_{m}^{k}}$$

where $S_{m}^{k}$ denotes HM signal intensity in the *k*-th genome region within the *m*-th sample. These genome regions include conserved H3K27ac regions, cancer-specific gained and lost H3K27ac regions, promoters (±2 kb around the transcription start site (TSS)), gene bodies (2 kb downstream of the TSS to the transcription termination site), and the first exons. $N_{m}$ and $N_{m}^{k}$ represent the total read counts and the read counts mapped into the *k*-th genome region in the *m*-th sample, respectively. $L_{m}^{k}$ describes the length of the *k*-th genome region.

#### 4.2.2. Chromatin Accessibility Profiling via DNase-Seq and ATAC-Seq

DNase-seq and ATAC-seq BAM files were converted to BED format by Bedtools [44]. Chromatin accessibility for predefined genomic regions in each sample was quantified using the method described in Equation (1). The results were then aggregated into a chromatin accessibility signal matrix. To reduce batch effects, the log_2_-transformed signal matrix underwent quantile normalization. For each predefined region, accessibility change was calculated as the difference in mean normalized signal between cancer and control samples. The distributions of signal differences were compared between conserved regions and lost or gained regions using two-sided Wilcoxon rank-sum tests (*p* < 0.05).

#### 4.2.3. Spatial Chromatin Architecture Analysis via Hi-C

To investigate the impact of H3K27ac dysregulation on three-dimensional (3D) chromatin organization, Hi-C datasets were processed using Juicer [47] to generate a Hi-C interaction matrix at a 5 kb resolution. As interaction frequency generally decreases with increasing genomic distance, we applied distance-based normalization as follows: (i) The genomic distance between bins *i* and *j* was defined as d=|i−j|×5kb. (ii) For a given genomic distance *d* on the same chromosome, we calculated the average interaction frequency as ${\bar{f}}_{d}=\frac{1}{b}\sum m_{ij}$, where *b* is the number of bin pairs separated by distance *d*. (iii) Each raw matrix entry was normalized by its corresponding average interaction frequency at distance *d*, calculated as $m_{ij}^{\prime }=m_{ij}/{\bar{f}}_{d}$. (iv) Based on these steps, the Hi-C matrix $M=\{m_{ij}\}$ was normalized into $M^{\prime }=\{m_{ij}^{\prime }\}$.

Following this normalization, we obtained distance-normalized Hi-C interaction matrices for cancer samples $T^{\prime }=\{t_{ij}^{\prime }\}$ and normal samples $N^{\prime }=\{n_{ij}^{\prime }\}$ for leukemia, BRCA, LUAD, and lymphoma, respectively. To evaluate chromatin interaction differences around the studied H3K27ac modification regions between cancer and normal samples at various genomic scales, we performed the following steps: (i) For the *k*-th H3K27ac region, we identified its corresponding bin pairs $t_{ij}^{\prime }$ and $n_{ij}^{\prime }$ in the Hi-C matrix by calculating $\left\lfloor x_{k}/5kb\right\rfloor \times 5kb$, where $x_{k}$ is the center coordinate of the *k*-th H3K27ac region; $\left\lfloor x_{k}/5kb\right\rfloor \times 5kb$ corresponds to the index of the *i*-th or *j*-th bin. (ii) We predefined a set of genomic distance thresholds *L* (*L* = 20 kb, 50 kb, 100 kb, 200 kb, 500 kb, and 1000 kb). For the *k*-th H3K27ac region, we collected all interaction values belonging to $\left\lfloor x_{k}/5kb\right\rfloor \times 5kb\pm L$. (iii) For each threshold *L*, changes in interaction frequency between cancer and normal samples were assessed for each H3K27ac region using a paired, two-tailed Student’s *t*-test, followed by Benjamini–Hochberg correction across all tested regions at that threshold, with significance defined as an FDR-adjusted *p*-value < 0.05 [48].

#### 4.2.4. RNA-Seq Data Analysis

For RNA-seq data from the ENCODE database, transcript abundance was quantified using Salmon against a human hg38 transcriptome reference [49]. Expression levels were reported in transcripts per million [50]. For leukemia RNA-seq data sourced from the GEO database, reannotation and quantification were carried out as described in our previous work [32].

### 4.3. Construction of H3K27ac Union Peak Sets

To identify highly conserved and cancer-specific H3K27ac modification regions, we drew on the computational framework developed by Fang et al. [20]. We first pooled all peaks called by MACS2 [43] across the 209 H3K27ac datasets and retained those with MACS2-derived enrichment scores ≥ 4 [20]. Because H3K27ac signals can span multiple nucleosomes, we examined the distribution of distances between adjacent peak summits to determine a representative peak width, denoted as *l*. Based on this width, we extended each summit by ±l/2 to establish the genomic boundaries of individual peaks. Finally, overlapping extended peaks were merged, and non-overlapping ones were retained, ultimately yielding a set of union H3K27ac-enriched regions.

### 4.4. Identification of Highly Conserved H3K27ac Modification Regions

For each H3K27ac union peak, we calculated its occupancy score and occupancy frequency. The occupancy score was defined as the number of H3K27ac ChIP-seq datasets harboring at least one filtered peak within the union peak. Since a total of 209 H3K27ac ChIP-seq datasets were analyzed, the occupancy score ranged from 1 to 209. The occupancy frequency was calculated as the ratio of the occupancy score to the total number of datasets. Union peaks with occupancy frequencies ≥ 80% (corresponding to presence in ≥168 datasets) were defined as conserved H3K27ac modification regions.

### 4.5. Recognition of Cancer-Specific Gained and Lost H3K27ac Modification Regions

To recognize cancer-specific gained and lost H3K27ac modification regions, we calculated the occupancy scores and occupancy frequencies of H3K27ac union peaks within each specified cancer, following the method described in Section 4.4. The LUAD, leukemia, HCC, BRCA, and lymphoma groups comprised 12, 8, 7, 8, and 24 cancer datasets, respectively, with 4 corresponding comparison datasets for each group (Supplementary Table S2). Subsequently, three criteria were adopted: (i) H3K27ac union peaks exhibited significantly higher (gained) or lower (lost) occupancy frequencies in specified cancer datasets compared to all datasets. (ii) H3K27ac signal intensity at each union peak was significantly elevated (gained) or reduced (lost) in specified cancers relative to all other datasets. Statistical significance was assessed using two-tailed independent *t*-tests and adjusted via the Benjamini–Hochberg method. (iii) To minimize tissue-specific effects, occupancy frequency and signal intensities were also compared between cancer samples and corresponding normal samples. Detailed identification criteria were as follows:

(1) **Gained regions**: (i) Occupancy frequency in the specified cancer ≥ $\left\lfloor 0.8\times c\right\rfloor$; *c* is the cancer sample number. (ii) Occupancy frequency across all datasets ≤ $\left\lfloor 0.2\times 209\right\rfloor$. (iii) Occupancy score = 0 in corresponding normal samples. (iv) H3K27ac signal intensities in cancer samples were significantly higher than those in all other datasets and their corresponding normal samples (*p*-value ≤ 0.01).
(2) **Lost regions**: (i) Occupancy frequency in the specified cancer ≤ $\left\lfloor 0.2\times c\right\rfloor$. (ii) Occupancy frequency across all datasets ≥ $\left\lfloor 0.8\times 209\right\rfloor$. (iii) Occupancy frequency in matched normal samples ≥ $\left\lfloor 0.5\times n\right\rfloor$; *n* is the normal sample number. (iv) H3K27ac signal intensities in the specified cancer were clearly lower than those in all other datasets and corresponding normal samples (*p*-value ≤ 0.01).

### 4.6. Definition of Genes Related to Cancer-Specific and Conserved H3K27ac Modification Regions

To define putative target genes related to conserved or cancer-specific H3K27ac modification regions, we calculated the genomic distance between the center of each H3K27ac region and the TSSs of all protein-coding genes. A gene was assigned as a target if the center of an H3K27ac region was located within the gene promoter (promoter-associated target) or within the gene body (body-associated target).

### 4.7. Transcription Factor Enrichment Analysis

TF enrichment analysis was conducted via BART [29], which uses a curated set of union DNase I hypersensitive sites (UDHSs) that represents a comprehensive catalog of candidate cis-regulatory elements across the genome. A total of 7968 ChIP-seq datasets for 918 TFs were compiled. For each TF, a binary profile was generated to indicate whether its ChIP-seq peaks overlap with at least one UDHS. Enrichment analysis was conducted by comparing TF binding frequency at cancer-specific H3K27ac-overlapped UDHSs with the frequency at all UDHSs. Statistical significance was evaluated using a two-tailed Fisher’s exact test.

### 4.8. Definition of Transcription Factor Target Genes

For a TF, we quantified TF–gene association strength (TFAS) as the weighted sum of TF binding peak intensities within ±20 kb of each gene’s TSS:

(2) $$TFAS=\sum i_{k}e^{-d_{k}/d_{0}}$$

where *i_k_* denotes the intensity of the *k*-th TF binding peak determined by MACS2. *d_k_* is the distance between the center of the *k*-th peak and the gene’s TSS. *d_0_* is a constant, set to 2000 bp [45,51].

After computing TFAS values, we applied a three-step filtering strategy to define TF target genes: (i) For the TF of interest, we averaged its TFAS values across all available ChIP-seq datasets and selected the top 100 genes with the highest values. (ii) For each individual dataset, we again selected the top 100 genes with the highest TFAS values and retained genes appearing in the top 100 list of at least three datasets. (iii) The target genes for the TF were defined as the intersection of genes identified in steps (i) and (ii).

## 5. Conclusions

In summary, this study establishes a high-resolution, multi-cancer atlas of H3K27ac modification regions, successfully decoupling malignant-identity-defining regulatory regions from highly conserved ones. Our integrative multi-omics analyses suggest that cancer-specific H3K27ac gains are associated with dynamic regulatory hubs characterized by oncogenic transcription factor recruitment and three-dimensional chromatin remodeling. By highlighting the GATA1–TAL1 axis as an example of this epigenetic–structural coupling, our findings provide a framework for understanding the potential relationship between local histone modifications and broader changes in chromatin organization. Furthermore, the CCHMR web server provides a translatable resource for the broader research community to prioritize functional regulatory elements in diverse biological contexts. Ultimately, our findings delineate a comprehensive landscape of the cancer epigenotype, offering a robust framework for the discovery of next-generation epigenetic biomarkers and therapeutic targets.

## Data Availability

All data used in this study are described in Section 4.1. All datasets analyzed in this study are publicly available from the GEO and ENCODE repositories, with accession numbers provided in the additional Supplementary Table S7. The CCHMR webserver is publicly accessible at https://www.imu-zgroup.com/CCHMR/ (accessed on 20 October 2026). All figures and tables in this manuscript are original and were generated by the authors based on publicly available datasets and in-house analyses; no content is reprinted or adapted from previously published works.

## Supplementary Materials

The following supporting information can be downloaded at https://www.mdpi.com/article/10.3390/ijms27219606/s1.
