# Supplementary material for: Deciphering Cancer-Specific H3K27ac Landscapes Through Systematic Multi-Omics Integration and Development of Open-Access Genomic Annotation Platform
Source: Int J Mol Sci. 2026 Oct 27;27(21):9606. doi: 10.3390/ijms27219606 (PMC13763514; doi:10.3390/ijms27219606)
Supplement: Supplementary file 1 [file ijms-27-09606-s001.zip › Supplementary file.pdf]

Deciphering Cancer-Specific H3K27ac Landscapes Through Systematic Multi-Omics Integration and Development of Open-Access Genomic Annotation Platform

Yu-Shuo Zhuang [1,†], Yuan-Yuan Gao [2,†], Zhao-Yue Zhang [3], Zhe-Peng Wang [1], Jun-Jie Liu [1], Li-Rong Zhang [1], Siqin Bateer [2,*] and Lu-Qiang Zhang [1,*]

[1] Inner Mongolia Key Laboratory of Biophysics and Bioinformatics, School of Physical Science and Technology, Inner Mongolia University, Hohhot 010021, China; 13734783903@163.com (Y.-S.Z.); tr3135582094@163.com (Z.-P.W.); pyljj@imu.edu.cn (J.-J.L.); pyzlr@imu.edu.cn (L.-R.Z.)

[2] Inner Mongolia Traditional Chinese & Mongolian Medical Research Institute, Hohhot 010020, China; 15849324447@163.com

[3] School of Medicine, University of Electronic Science and Technology of China, Chengdu 610054, China; zyzhang@uestc.edu.cn

* Correspondence: bateer_s@imu.edu.cn (S.B.); 111989025@imu.edu.cn (L.-Q.Z.)

† These authors contributed equally to this work.

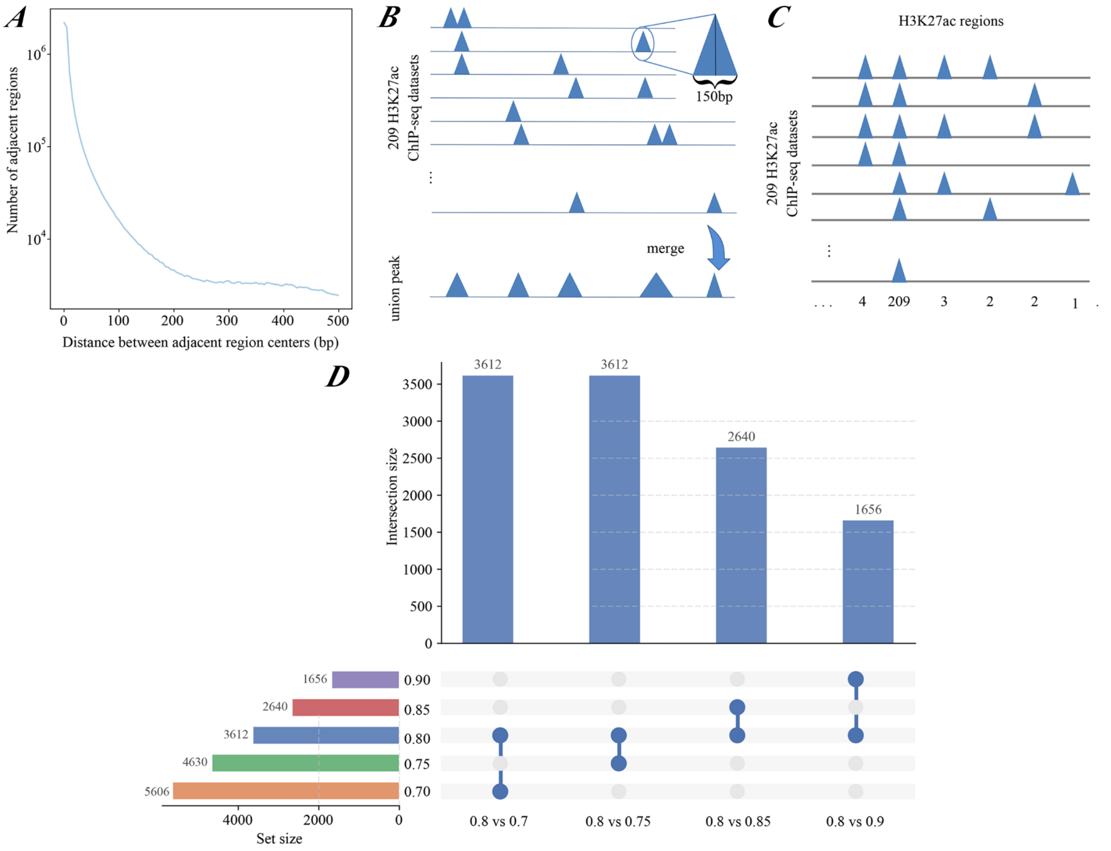

Figure S1. Identification of conserved H3K27ac-modified regions. (A) Distribution of distances between adjacent H3K27ac peaks. (B) Schematic representation of H3K27ac union peak merging. Peaks with summit distances ≤150 bp were merged, resulting in 678,446 non-overlapping H3K27ac union peaks. (C) Definition of occupancy score for each union peak across 209 ChIP-seq datasets. (D) Conserved H3K27ac region sets and their intersections across five thresholds, centered on the 0.8 baseline. Left bars show the number of conserved regions at each threshold (0.7, 0.75, 0.8, 0.85, 0.9). Right bars indicate intersections with the 0.8 baseline.

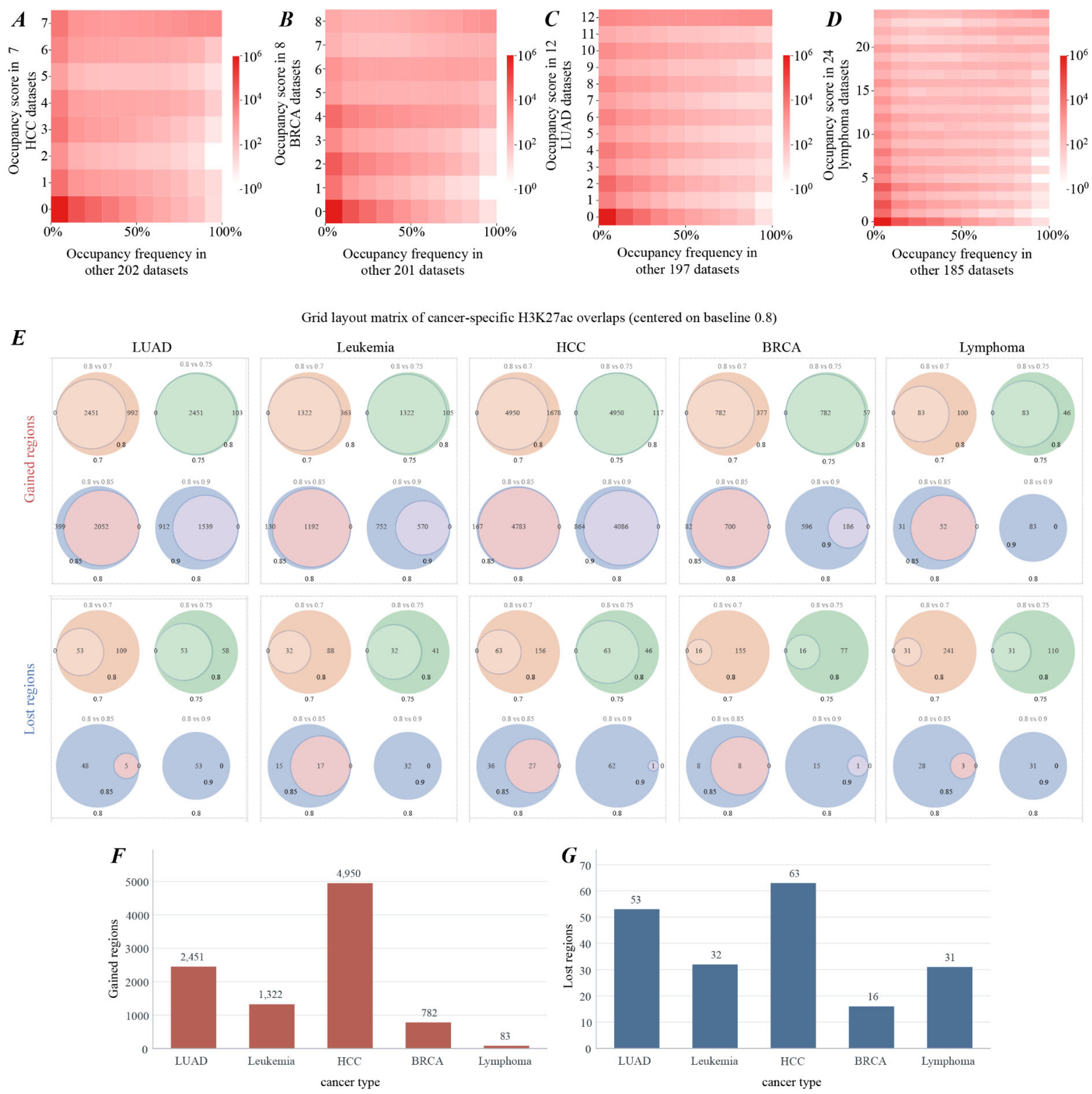

Figure S2. Identification of cancer-specific regions. (A-D) The occupancy score distribution of 678,446 union peaks in four specified cancer datasets (y-axis) plotted against their occupancy frequencies in all remaining datasets. Color intensity corresponds to the number of peaks. (E) Overlap matrix of cancer-specific H3K27ac regions across thresholds. Rows: gained/lost regions; columns: five cancer types (LUAD, Leukemia, HCC, BRCA, Lymphoma). Each cell contains four Venn diagrams comparing the baseline cutoff (0.8, blue) with 0.7, 0.75, 0.85, and 0.9 (orange, green, red, purple). (F-G) Numbers indicate region counts in each subset. Numbers of cancer-specific gained and lost H3K27ac regions identified in each of the five cancer types.

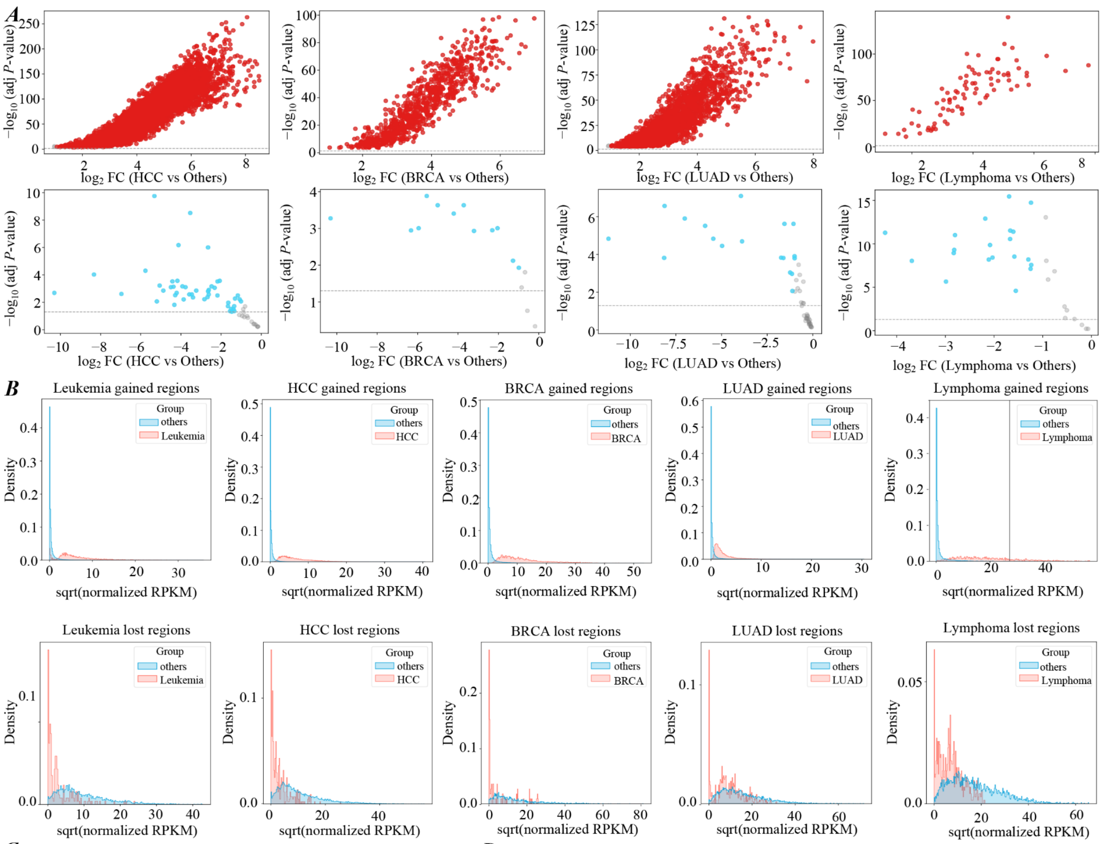

C

	effect size of lost regions	effect size of gained regions
Leukemia	−0.94	3.46
HCC	−0.77	3.88
BRCA	−0.73	3.80
LUAD	−0.59	2.05
Lymphoma	−0.95	3.13

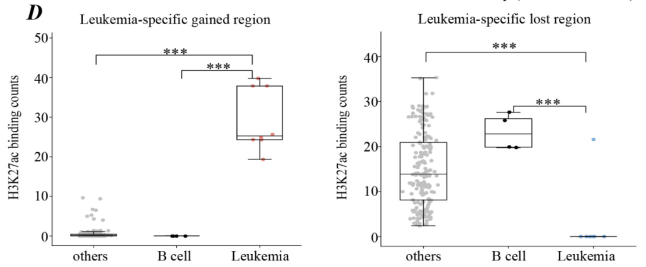

Figure S3. Differential H3K27ac modification patterns in cancer-specific gained and lost regions across four cancer types. (A) H3K27ac signal enrichments in each of four cancer samples relative to all other samples for gained (top) and lost (bottom) regions. Each point represents a genomic region, the x-axis indicates the $\log_2$ fold change in H3K27ac signal intensity and the y-axis shows the -$\log_{10}$-transformed p-value. The dashed line marks the significance threshold (p-value=0.05). (B) Distributions of H3K27ac signals at cancer-specific gained (top) and lost (bottom) regions in corresponding cancer samples (y-axis) compared with their distributions in all remaining samples. X-axis indicates the quantile-normalized H3K27ac signals. (C) Effect sizes of cancer-specific gained and lost H3K27ac regions across five cancer types. (D) A representative example showing the distributions of H3K27ac read counts at leukemia-specific gained (red) and lost (blue) regions in leukemia samples, the corresponding normal samples (black), and other samples (grey). Statistical significance: p-value < 0.05 (*), p-value < 0.01 (**), p-value < 0.001 (***).

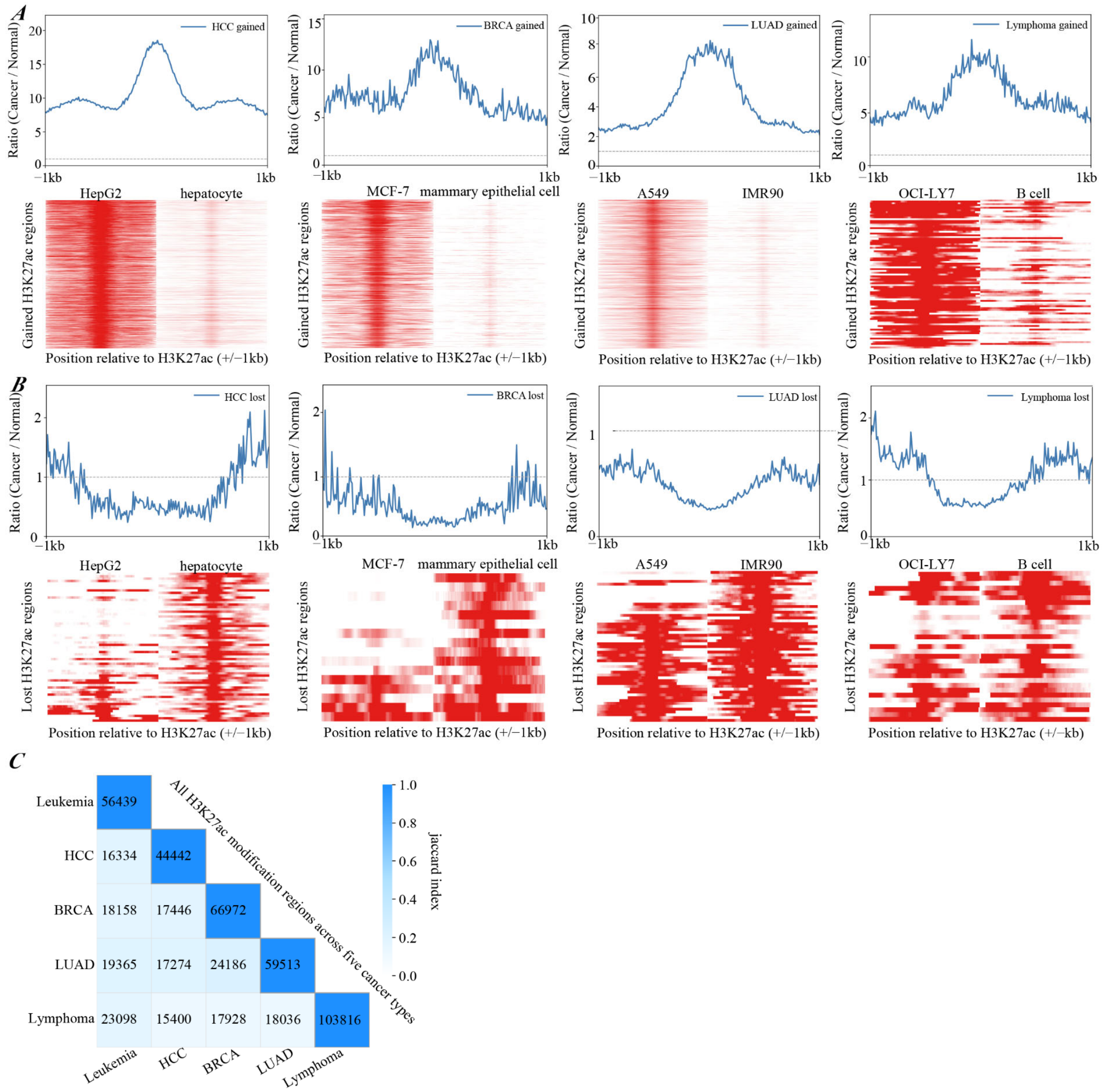

Figure S4. H3K27ac signal profiles and regional overlap across different cancer types. (A, B) H3K27ac ChIP-seq signal intensities (±1 kb) centered on cancer-specific gained (A) and lost regions (B) in four distinct cancer samples and their corresponding normal samples. Line plots (above) show the average ratios of cancer to normal signal intensities; the dashed line represents a ratio of 1. (C) Number and pairwise overlap of all identified H3K27ac binding regions across the five cancer types. Color scale represents Jaccard similarity, with darker shades indicating greater overlap.

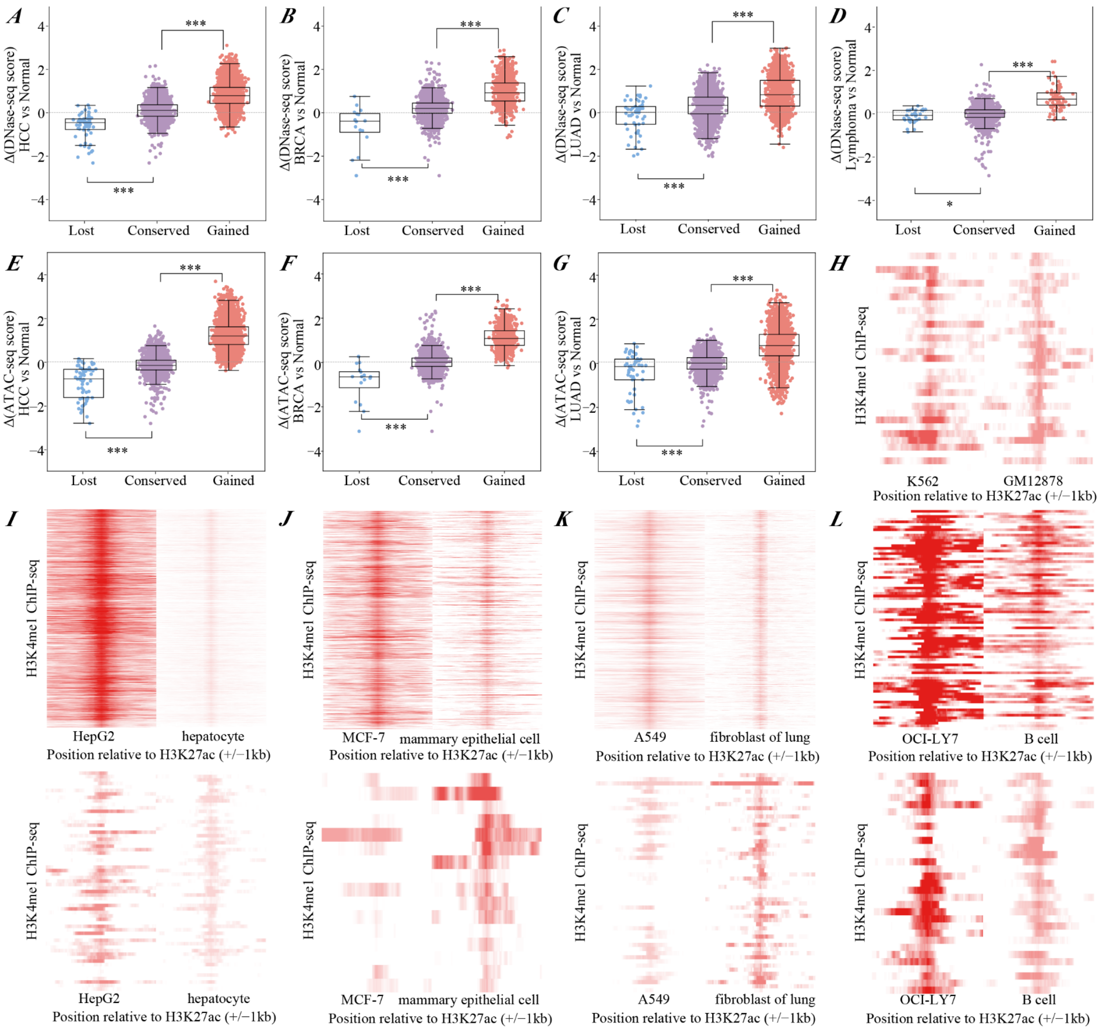

Figure S5. Chromatin accessibility and enhancer-associated histone mark dynamics at conserved and cancer-specific H3K27ac regions across multiple cancer types. (A-D) Dynamic landscapes of DNase-seq signal intensities in conserved and cancer-specific gained and lost H3K27ac regions across HCC, BRCA, LUAD and lymphoma. (E-G) Dynamic landscapes of ATAC-seq signal intensities in conserved and cancer-specific gained and lost H3K27ac regions across HCC, BRCA and LUAD. (H-L) H3K4me1 ChIP-seq signal intensities within ±1 kb of the center of cancer-specific lost H3K27ac regions across leukemia, HCC, BRCA, LUAD and lymphoma. (M-N) H3K4me1 ChIP-seq signal intensities within ±1 kb of the center of cancer-specific gained H3K27ac regions across HCC, BRCA, LUAD and lymphoma.

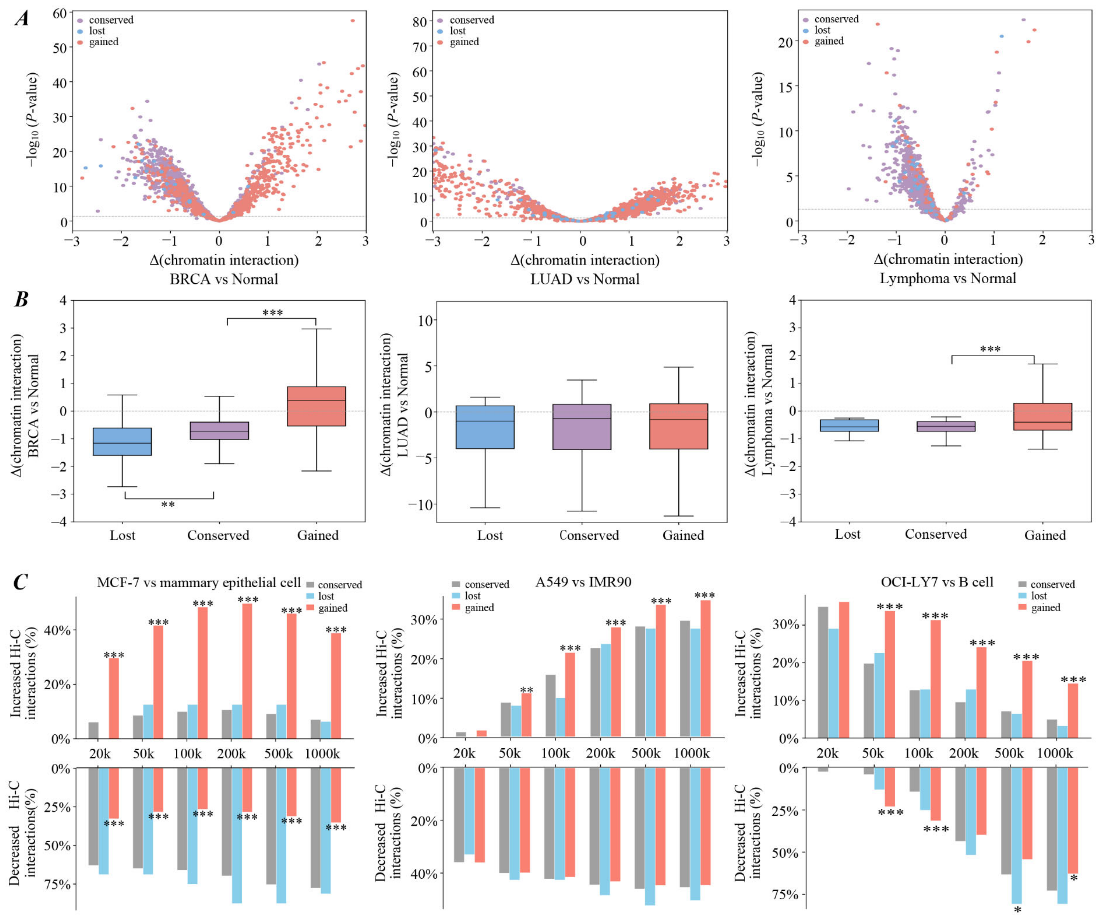

Figure S6. Cancer-specific H3K27ac regions are associated with altered chromatin interaction patterns. (A) Differential chromatin interaction levels between cancer and normal samples across the three H3K27ac regions in BRCA, LUAD and lymphoma, as quantified by Hi-C. Each point represents the interaction changes between a H3K27ac region and 5-kb bins located within 500 kb of its center. (B) Differential interaction frequencies between BRCA, LUAD and lymphoma samples and their corresponding normal samples across the three H3K27ac regions. (C) Proportions of conserved (grey) and cancer-specific lost (blue) and gained (orange) regions exhibiting increased (top) or decreased (bottom) local chromatin interactions in BRCA, LUAD and lymphoma samples relative to corresponding normal controls. Local interactions are defined as contacts between H3K27ac modification regions and 5kb bins within 20kb, 50kb, 100kb, 200kb, 500kb, 1000kb, respectively. The chromatin interaction change between cancer and normal samples was assessed using groupwise average comparisons. Per-region paired t-test p-values were adjusted using the Benjamini Hochberg FDR method. Statistical significance was assessed using FDR-adjusted p-values (**p*-value < 0.05, ***p*-value < 0.01, ****p*-value < 0.001).

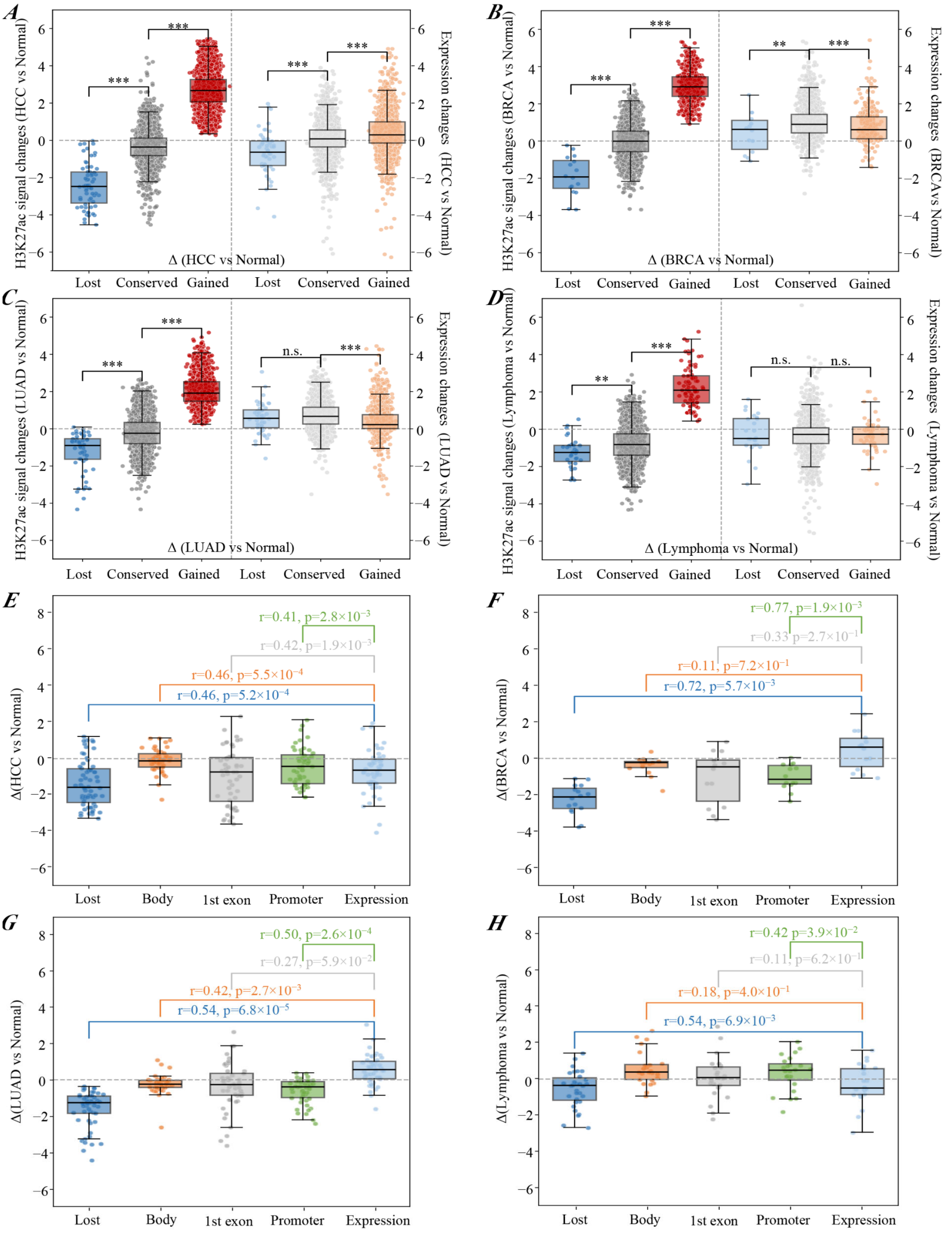

Figure S7. Cancer-specific H3K27ac modification events are linked to gene expression changes during tumorigenesis. (A-D) Comparative analysis of H3K27ac signal intensity changes (left) and corresponding gene expression changes (right) across conserved regions, cancer-specific gained and lost regions in HCC, BRCA, LUAD and lymphoma. (E-H) Pearson correlation analysis between H3K27ac signal intensity changes in different functional regions and the corresponding gene expression alterations in HCC, BRCA, LUAD and lymphoma.

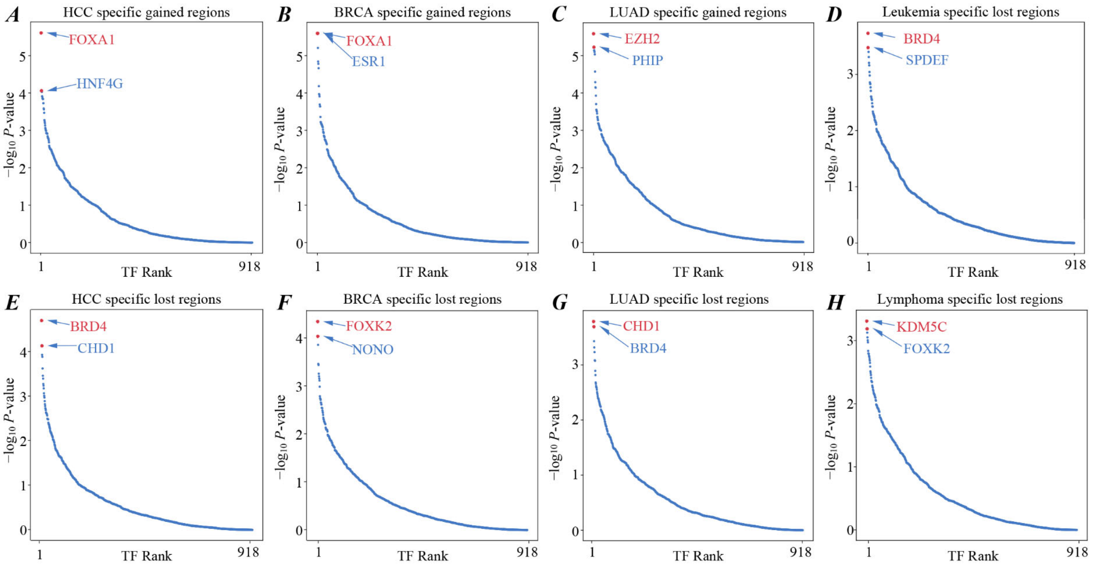

Figure S8. Key transcription factors predicted by BART to be enriched in cancer-specific gained and lost H3K27ac regions.

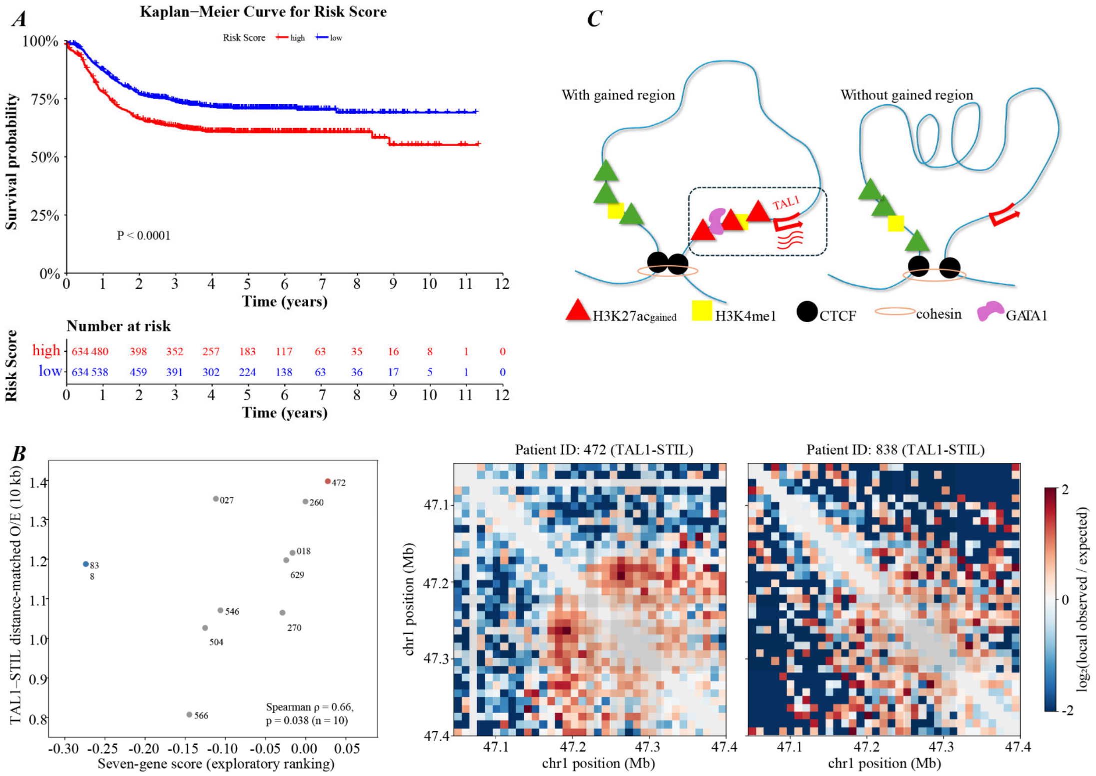

Figure S9. Clinical relevance and chromatin regulatory mechanism of the H3K27ac–GATA1–TAL1 axis in leukemia. (A) Kaplan-Meier overall survival curves for high-risk and low-risk patients in the TARGET cohort. (B) Chromatin interactions at the TAL1-STIL locus in high-risk and low-risk leukemia patients. (C) Proposed model linking leukemia-specific H3K27ac gain to GATA1 binding and TAL1 activation.
